# Reviewer Acknowledgement 2013

**DOI:** 10.1186/1471-2474-15-15

**Published:** 2014-01-16

**Authors:** Catia Cornacchia

**Affiliations:** 1 BioMed Central, Floor 6, 236 Gray's Inn Road, London, WC1X 8HB, United Kingdom

## Abstract

**Contributing Reviewers:**

The editors of *BMC Musculoskeletal Disorders* would like to thank all our reviewers who have contributed to the journal in Volume 14 (2013).

## 

Aaron Nauth

Canada

Adam Carroll

Australia

Adam Shortland

UK

Adam Goode

USA

Adam Garrow

UK

Adriaan O Grobbelaar

UK

Adriana Soares

Brazil

Adriana Ioachimescu

USA

Ahmad Addosooki

Egypt

Ailsa Welch

UK

Aisha Siddiqah Ahmed

Sweden

Akio Kobayashi

Japan

Ákos Kynsburg

Austria

Alan Tennant

UK

Alberto Migliore

Italy

Alberto Tagliafico

Italy

Albrecht Popp

Switzerland

Alex Burdorf

Netherlands

Alexander Auffarth

Austria

Alexander Liddle

UK

Alexander Ruhe

Germany

Alexander Green

UK

Alexander Theodorus Maria Van De Water

Australia

Alexandre Lädermann

Switzerland

Alexandrina Mendes

Portugal

Alexej Barg

Switzerland

Alexis Wright

USA

Alfonso Manzotti

Italy

Ali Montazeri

Iran

Ali Fahir Ozer

Turkey

Aliah Shaheen

UK

Aline Ramond-Roquin

France

Alma Becic Pedersen

Denmark

Alvin Ong

USA

Ambreen Chohan

UK

Amr Sawalha

USA

Ana Isabel Carita

Portugal

Ana-Maria Vranceanu

USA

Anders Enocson

Sweden

Andreas Mavrogenis

Greece

Andreas Winkelmann

Germany

Andreea Ioan

Netherlands

Andrej Maria Nowakowski

Switzerland

Andrew Phillips

UK

Andrew Sprowson

UK

Andrew Briggs

Australia

Andrew Blann

UK

Andrew Claus

Australia

Andrew Kittelson

USA

Andrew Östör

UK

Andrzej Zyluk

Poland

Angel Checa

USA

Angela Colantonio

Canada

Angela Evans

Australia

Angelica Gattamelata

Italy

Angelo Cacchio

Italy

Anita Williams

UK

Anna Bonjoch

Spain

Annalisa Monaco

Italy

Anne Daly

Australia

Anne Gingery

USA

Anne Smith

Australia

Anne Juvani

Finland

Anne-Kathrin Tausche

Germany

Anneli Ojajärvi

Finland

Annelie Weinberg

Austria

Anne-Maree Keenan

UK

Annemieke Boot

Netherlands

Annemieke Aartsma-Rus

Netherlands

Annet Van Rijssen

Netherlands

Anthony Perruccio

Canada

Antonia Chen

USA

Antonia Gómez Conesa

Spain

Antonio Julià

Spain

Antonio Herrera

Spain

Antonio Gonzalez

Spain

Antonio Spadaro

Italy

Antonio Fernandez-Nebro

Spain

Antonio La Cava

USA

Arjen Blom

Netherlands

Arnaud Dupeyron

France

Arnd Hoburg

Germany

Arne Fischmann

Switzerland

Aron Downie

Australia

Arthur Lau

Canada

Ashok Gavaskar

India

Astrid Woodhouse

Norway

Atsuo Nakamae

Japan

Axel Gamulin

Switzerland

Axel Skytthe

Denmark

Berend Willem Schreurs

Netherlands

Bahar Sadeghi Abdollahi

Iran

Barbara Nicholl

UK

Barbara Yawn

USA

Barbara Rossi

Italy

Bård Natvig

Norway

Bart Visser

Netherlands

Bart Koes

Netherlands

Bart Clarke

USA

Beatriz Carames Perez

USA

Behzad Heidari

Iran

Bela Melegh

Hungary

Benjamin Fernandez-Gutierrez

Spain

Benjamin Fisher

UK

Bérengère Aubry-Rozier

Switzerland

Beril Talim

Turkey

Berit Grandaunet

Norway

Bernadett Balla

Hungary

Bernard Cortet

France

Bernd Markert

Germany

Berta Cillero Pastor

Netherlands

Beth Fox

USA

Bianca Bignotti

Italy

Bing Lu

USA

Binu Thomas

India

Birgit Greiner

Ireland

Birgit Juul-Kristensen

Denmark

Birthe Thomsen

Denmark

Bita Shakoory

USA

Bjorgulf Claussen

Norway

Bjorn Gerdle

Sweden

Björn Salomonsson

Sweden

Bjørn Skallerud

Norway

Borysov Maksym

Ukraine

Brady Tripp

USA

Brandon Santoni

USA

Brenda Monaghan

Ireland

Brent Baker

USA

Brent Leininger

USA

Brett Vaughan

Australia

Brett Levine

USA

Brian Minnema

Canada

Brian Pietrosimone

USA

Bronek Boszczyk

UK

Brunella Grigolo

Italy

Bruno Laganá

Italy

Burel Goodin

USA

Byron Hoogwerf

USA

Cayetano Alegre

Spain

Carl Lauryssen

USA

Carl Lombard

South Africa

Carlo Perricone

Italy

Carlo Cosimo Quattrocchi

Italy

Carlos Rodrigues

Brazil

Carlos J. Marques

Germany

Caroline Hoemann

Canada

Caroline Bastiaenen

Netherlands

Caroline Flurey

UK

Caroline Sanders

UK

Caroline Smith

USA

Caroline Hing

UK

Cathy Holt

UK

Cemal Kazimoglu

Turkey

Cemil Kayali

Turkey

Cesar Fernandez-De-Las

Spain

Cha Guochun

China

Chalotte Beaudart

Belgium

Chamith Rajapakse

USA

Chang-Fu Kuo

UK

Chang-Qing Zhang

China

Chao Xie

USA

Charalampos Papagoras

Greece

Charlie Hicks-Little

USA

Charlotte Leboeuf-Yde

Denmark

Cheryl Hawk

USA

Cheryl Metcalf

UK

Chi-Chuan Wu

Taiwan

Chih-Hsiu Cheng

Taiwan

Ching-Chi Hsu

Taiwan

Ching-Lung Tai

Taiwan

Chong Bum Chang

South Korea

Chris Recknor

USA

Chris Littlewood

UK

Christian Heisel

Germany

Christian Woiciechowsky

Germany

Christian Juergens

Germany

Christian Merle

Germany

Christian Dejaco

Austria

Christiana Savvidou

USA

Christina Lampe

Germany

Christina Ahlgren

Sweden

Christina Gummesson

Sweden

Christina Pelajo

Brazil

Christine Cedraschi

Switzerland

Christine Goertz

USA

Christine Novak

Canada

Christoph Raschka

Germany

Christy Tomkins-Lane

Canada

Chunfeng Zhao

USA

Cionne Manning

USA

Clare Jinks

UK

Claudia Giacomozzi

Italy

Claudia Cicione

Italy

Claudio Colosio

Italy

Claudio Dora

Switzerland

Claus Manniche

Denmark

Clayton Peimer

USA

Cliona Mcrobert

UK

Colleen Mchorney

USA

Costantino Errani

Italy

Craig Schulz

USA

Cy Frank

Canada

Cynthia Sieck

USA

Cyril Mauffrey

USA

Daichi Hayashi

USA

Dan Riddle

USA

Dana Bliuc

Australia

Dana Lawrence

USA

Dana Judd

USA

Daniel Espino

UK

Daniel Solomon

USA

Daniel Bossen

Netherlands

Daniel White

USA

Daniel Mcwilliams

UK

Daniel Fong

China

Daniel Kendoff

Germany

Danielle Van Der Windt

UK

Danijela Djonic

Serbia

David Torgerson

UK

David Bichara

USA

David Gould

UK

David Felson

USA

David Krause

USA

David Rempel

USA

David Newell

UK

David Mcbride

New Zealand

David Walton

Canada

David Hak

USA

David Stroh

USA

David Ring

USA

David Behm

Canada

David Coggon

UK

David Sheps

Canada

Davide Malatesta

Switzerland

Davide Maria Donati

Italy

Dawn Stacey

Canada

Dawn Dowding

UK

Deborah Kado

USA

Deepak Goyal

India

Dehao Fu

China

Dennis Anderson

USA

Deva Chan

USA

Dhanasekara Raja Palanisami

India

Diane Jette

USA

Diane Gyi

UK

Dimitri Popkov

Russia

Dinesh Thawrani

USA

Dirk Jan Moojen

Canada

Dominic Southgate

UK

Dominic Furniss

UK

Donal Mcnally

UK

Dongquan Shi

China

Donna Urquhart

Australia

Douglas Wardlaw

UK

Duncan Reid

New Zealand

Edward Davis

UK

Egon Perilli

Australia

Eijiro Maeda

Japan

Eileen Britt

New Zealand

Elaine Karis

USA

Elisabeth Andreadou

Greece

Elizabeth Barrett-Connor

USA

Elizaveta Kon

Italy

Elsa Strotmeyer

USA

Else Marie Bartels

Denmark

Emma Stanmore

UK

Emma Healey

UK

Ennio Lubrano

Italy

Enrique Guerado

Spain

Eric Levicoff

USA

Eric Farrell

Netherlands

Erik Fink Eriksen

Norway

Erik Rader

USA

Erik Taal

Netherlands

Erik L. Werner

Norway

Esa Jämsen

Finland

Esther Williamson

UK

Eswar Krishnan

USA

Eva Vingård

Sweden

Eyal Itshayek

Israel

Fabian Stuby

Germany

Fabio Galbusera

Italy

Fabiola Atzeni

Italy

Fackson Mwale

Canada

Fahad Alkherayf

Canada

Faisal Latif

USA

Farzam Farahmand

Iran

Federica Ginanneschi

Italy

Federico Canavese

France

Federico Balagué

Switzerland

Fernando Martins

Portugal

Fhr De Man

Netherlands

Fiona Webster

Canada

Fiona Paul

UK

Fiona Wilson

Ireland

Flavia Cicuttini

Australia

Florence Tsui

Canada

Florian Gebhard

Germany

Frances Williams

UK

Francesc Cots

Spain

Francis Berenbaum

France

Francisco Alburquerque-Sendín

Spain

Francisco O'Valle

Spain

Franco Antoniazzi

Italy

Franco Postacchini

Italy

Francois Fourchet

Qatar

Frank Sloan

USA

Frank Handelberg

Belgium

Frank Roemer

Germany

Freeman Miller

USA

Friederike Kendel

Germany

Fulvia Ceccarelli

Italy

Gabriel Piza Vallespir

Spain

Gabriel Herrero-Beaumont

Spain

Gabrielle Tuijthof

Netherlands

Gareth John Treharne

New Zealand

Geoff Bostick

Canada

Geoff Littlejohn

Australia

Georg Mattiassich

Austria

Georg Osterhoff

Switzerland

Georg Neumann

Germany

Georgia Ntani

UK

Georgios Filippou

Italy

Georgios Belibasakis

Switzerland

Gerard Slobogean

Canada

Gerhard Sengle

Germany

Gerjo Van Osch

Netherlands

Gertraud Gradl

Germany

Gethin Thomas

Australia

Ghassan Maalouf

Lebanon

Gioacchino Li Cavoli

Italy

Giorgio Tasca

Italy

Giorgio Della Rocca

Italy

Giorgio Salvi

Italy

Giovanni Merolla

Italy

Girdhar Agarwal

India

Girija Rath

India

Giuseppe Solarino

Italy

Giuseppe Granata

Italy

Glen Mervyn Blake

UK

Glinda Cooper

USA

Godard De Ruiter

Netherlands

Grace Szeto

Hong Kong

Graeme Jones

Australia

Grant Townsend

Australia

Greg Kawchuk

Canada

Gregory Bain

Australia

Gregory Chang

USA

Guang-Hua Lei

China

Guangpeng Liu

China

Guglielmo Trovato

Italy

Guido Fitze

Germany

Gulcan Gurer

Turkey

Gundula Schulze-Tanzil

Germany

Guoan Li

USA

Gustavo Zanoli

Italy

Guy Trudel

Canada

Gwendolyn Sowa

USA

Gwenllian Wynne-Jones

UK

H. Thomas Temple

USA

Hairong Zheng

China

Hal Martin

USA

Hamidreza Pazoki Toroudi

Iran

Hanane Bahouq

Morocco

Haner Direskeneli

Turkey

Hannah Radley-Crabb

Australia

Hanno Millesi

Austria

Hans Savelberg

Netherlands

Hany Hefny

Egypt

Harmen Ettema

Netherlands

Harpreet Sangha

Canada

Harri Hamalaine

Finland

Harriet Wittink

Netherlands

Harry Von Piekartz

Germany

Haruhiko Akiyama

Japan

Harukazu Tohyama

Japan

Hassan Ghomrawi

USA

Hatice Bodur

Turkey

Helen Gruber

USA

Helen Harcombe

New Zealand

Helena Canhao

Portugal

Helene M. Paarup

Denmark

Henrica De Vet

Netherlands

Henrik Hein Lauridsen

Denmark

Hermien Kan

Netherlands

Hilde Stendal Robinson

Norway

Hilmy Ismail

Australia

Hiroyuki Oka

Japan

Hiske Van Duinen

Sweden

Hitesh Modi

India

Hon Yuen

USA

Hossein Negahban

Iran

Howard Vernon

Canada

Hugo Giambini

USA

Huub Van Der Heide

Netherlands

Ian Stokes

USA

Ignacio Rego-Perez

Spain

Ilkka Helenius

Finland

Ingrid Sivesind Mehlum

Norway

Irene Szijan

Argentina

Isabel Andia

Spain

Isam Atroshi

Sweden

Ivan Lin

Australia

J. Bart Staal

Netherlands

J. Christoph Katthagen

Germany

Jack Crosbie

Australia

Jack Anavian

USA

Jacqueline Mair

UK

Jae-Do Kim

South Korea

Jae-Young Lim

South Korea

Jagdeep Nanchahal

UK

James Devocht

USA

James Mcauley

Australia

James Harrop

USA

James Fitzsimmons

USA

James Selfe

UK

James Fick

Finland

James Dowling

Canada

Jan Kool

Switzerland

Jane Zochling

Australia

Jan-Erik Gjertsen

Norway

Janice Lin

USA

Janina Patsch

USA

Jan-M. Brandt

Canada

Jannike Øyen

Norway

Jan-Paul Van Wingerden

Netherlands

Jari Ylinen

Finland

Jari Nuutila

Finland

Jay Hertel

USA

Jean Wong

Canada

Jean-François Cazeneuve

France

Jean-Pascal Lefaucheur

France

Jean-Pierre Estebe

France

Jeffrey Driban

USA

Jeffrey Katz

USA

Jennifer Wolf

USA

Jennifer Walsh

UK

Jennifer Barton

USA

Jens Walldorf

Germany

Jeremy Crenshaw

USA

Jerzy Jablecki

Poland

Jesper Knoop

Netherlands

Jessica Burke

USA

Jessica Deneweth

USA

Jiandong Hao

USA

Jiang Peng-Fei

China

Jianning Zhao

China

Jianying Zhang

USA

Ji-Hoon Bae

South Korea

Jill Waalen

USA

Jim Richards

UK

Jingming Xie

China

Jinn Lin

Taiwan

Jiunn-Horng Kang

Taiwan

Joan Cid

Spain

Joaquina Montilla-Herrador

Spain

Job Van Susante

Netherlands

Jochen Obernauer

Austria

Jochen Baumeister

Germany

Joel Patterson

USA

Joerg Boehme

Germany

Joerg Holstein

Germany

Johan Hviid Andersen

Denmark

Johanne Martel-Pelletier

Canada

Johannes Bussmann

Netherlands

Johannes Nossent

Norway

Johannes Antonius Van Der Sluijs

Netherlands

John Loughlin

UK

John Skedros

USA

John Kalbfleisch

USA

John O'Dowd

UK

Jonathan Hill

UK

Jonathan Adachi

Canada

Jonathan Sembrano

USA

Jonathan Jeffers

UK

Joop Van Den Bergh

Netherlands

Jorge Lopez Ayerbe

Spain

Jorge A. Roman-Blas

USA

Jose Moncada-Jimenez

Costa Rica

Jose A. Riancho

Spain

Jose Manuel Quesada Gomez

Spain

Josef Klocker

Austria

Joseph Dias

UK

Joseph Hamill

USA

Joshua Baker

USA

Juan Fernandez Tajes

Spain

Juan Concha

Colombia

Judith Sluiter

Netherlands

Jukka Ristiniemi

Finland

Julia Hush

Australia

Julia Treleaven

Australia

Junxiu Liu

USA

Junya Ozawa

Japan

Jurandir Nadal

Brazil

Jürgen Paletta

Germany

Kabul Saikia

India

Kai Cao

China

Kaisa Mannerkorpi

Sweden

Kang Li

USA

Kara Holloway

Australia

Karel Pavelka

Czech Republic

Karen Søgaard

Denmark

Karen Troy

USA

Karen Barker

UK

Kathryn Swoboda

USA

Katja Heinemeier

Denmark

Kazuya Tamai

Japan

Kazuyoshi Uchihashi

Japan

Kwok Chuen Wong

Hong Kong

Kees Boersma

Netherlands

Keiichi Kuroki

USA

Keijo Mäkelä

Finland

Keith Baldwin

USA

Keith Rome

New Zealand

Kelli Allen

USA

Kellie Stockton

Australia

Kelly Mcnagny

Canada

Kelsey Hegarty

Australia

Kengo Yamamoto

Japan

Kenneth Jay Andersen

Denmark

Kenton Kaufman

USA

Kevin Mulhall

Ireland

Kevin Perry

USA

Kevin Plancher

USA

Kheng Lim Goh

Singapore

Kieran O'Sullivan

Ireland

Kimieru Ito

Japan

Kimme Hyrich

UK

Kiriakos Daniilidis

Germany

Kirti Singh

India

Kiyohito Naito

Japan

Koichi Shinkoda

Japan

Koichi Masuda

USA

Kook Jin Chung

South Korea

Koonstantinos Natsis

Greece

Kourosh Zarghooni

Germany

Kristina Akesson

Sweden

Krysia Dziedzic

UK

Kun Zhu

Australia

Kwang Am Jung

South Korea

Kyoji Ikeda

Japan

Kyoung Hwan Koh

South Korea

Lalit Maini

India

Lance Mccracken

UK

Lars Großterlinden

Germany

Lars Gerhardsson

Sweden

Lars L. Andersen

Denmark

Laura Laslett

Australia

Laura Scaramuzzo

Italy

Laura Coates

UK

Lauren Beaupre

Canada

Laurence Marshman

Australia

Leena Mirjam Kaila-Kangas

Finland

Leigh F. Callahan

USA

Leonardo Costa

Brazil

Leonardo Punzi

Italy

Leslie Harrold

USA

Leslie Nicholson

Australia

Lexie Holliday

USA

Lilian Plotkin

USA

Lin Nie

China

Linda Li

Canada

Linda Popplewell

UK

Linda A. Dahlgren

USA

Lindsay Hickerson

USA

Lindsey Hooper

UK

Lisa Langsetmo

Canada

Lisbet Haglund

Canada

Lise Hestbaek

Denmark

Livia Puljak

Croatia

Ljubica Konstantinovic

Serbia

Lorena Longato

Italy

Lorin Michael Benneker

Switzerland

Louise Reynard

UK

Lourdes Basurto

Mexico

Luc De Smet

Belgium

Luca Pietrogrande

Italy

Luca Maria Sconfienza

Italy

Luciola Menezes-Costa

Brazil

Luigi Tarallo

Italy

Luis Catoggio

Argentina

Luis Alvarez

Spain

Luiz Garcez Leme

Brazil

Lukas Konstantinidis

Germany

Lynn Snyder-Mackler

USA

M John G Bankart

UK

Ma'Ad Alsaati

Canada

Maarit Piirtola

Finland

Maciej Buchowski

USA

Magali Cucchiarini

Germany

Maha El Gaafary

Egypt

Mahmood Khosrowjerdi

Iran

Malinee Laopaiboon

Thailand

Mami Tamai

Japan

Manabu Ito

Japan

Manuel Lemos

Portugal

Manuel Hernandez

USA

Manuela Glattacker

Germany

Manuela Ferreira

Armenia

Marc Moramarco

USA

Marc Levesque

USA

Marc Röllinghoff

Germany

Marcel Betsch

USA

Marcel Roy

USA

Marcella Neri

Italy

Marco Paoloni

Italy

Marco Matucci Cerinic

Italy

Marcus Schiltenwolf

Germany

Marcus Egermann

Germany

Maria Lopez-Armada

Spain

Maria Stoenoiu

Belgium

Maria Inacio

USA

Maria Del Carmen De Andres

UK

Maria Teresa Terreri

Brazil

Marianne Wallis

Australia

Mariano Fernandez-Fairen

Spain

Marie Birk Jorgensen

Denmark

Marie-Christophe Boissier

France

Marike Van Der Leeden

Netherlands

Mario Cabraja

Germany

Mario D. Cordero

Spain

Marios Lykissas

USA

Mark Eidelman

Israel

Mark Hancock

Australia

Mark Cooper

UK

Mark Tarnopolsky

Canada

Mark Lidegaard

Denmark

Markus Melloh

Australia

Markus Stuecker

Germany

Maros Hrubina

Czech Republic

Marsha Raebel

USA

Marsha Tijssen

Netherlands

Martha Cammarata

USA

Martin Descarreaux

Canada

Martin Mackey

Australia

Martin Stoddart

Switzerland

Martin Underwood

UK

Martin Quirno

USA

Martin L Verra

Switzerland

Martine Cohen-Solal

UK

Maruti Gudavalli

USA

Mary Barbe

USA

Mary O' Keeffe

Ireland

Masaaki Chazono

Japan

Masahiko Nozawa

Japan

Masahiro Hasegawa

Japan

Massimo Innocenti

Italy

Matilde Tschon

Italy

Matthew E. R. Butchbach

USA

Matthias Lerch

Germany

Matthias Schulz

Germany

Maurice Balke

Germany

Maurizio Benucci

Italy

Mauro Mondelli

Italy

Maximilian Rudert

Germany

Megan Bohensky

Australia

Mehmet Aydogan

Turkey

Mehmet Erdil

Turkey

Mei Zhu

China

Melissa Kacena

USA

Mercè Giner

Spain

Merrill Landers

USA

Michael Adams

UK

Michael Carmont

UK

Michael Bloomfield

USA

Michael Zlowodzki

USA

Michael Lee

USA

Michael Briggs

UK

Michael Yelland

Australia

Michael Nurmohamed

Netherlands

Michael Gardner

USA

Michael Davidsen

Denmark

Michael Reiman

USA

Michael Yin

USA

Michael Von Korff

USA

Michael Mienaltowski

USA

Michael Nissen

Switzerland

Michael A. Mont

USA

Michael Christian Liebensteiner

Austria

Michael G. Zywiel

Canada

Michaela Endres

Germany

Michal Polguj

Poland

Michel Le Duff

USA

Michel D. Crema

Brazil

Michele Maiers

USA

Michiel Reneman

Netherlands

Miguel Angel Ruiz-Ibán

Spain

Miho Sekiguchi

Japan

Min Tian

USA

Minna Mannikko

Finland

Minos Tyllianakis

Greece

Mirjana Kocic

Serbia

Mitchell Haas

USA

Mohamed Kenawey

Egypt

Mohammad Mansournia

Iran

Mohammed Akhter

USA

Mohit Bhandari

Canada

Mohsen Karami

Iran

Monique Ardon

Netherlands

Moritz Tannast

Switzerland

Morten Wærsted

Norway

Mustafa Younis

USA

Mwidimi Ndosi

UK

Nadine Hollevoet

Belgium

Naffis Anjarwalla

UK

Nageswara Koneti

India

Naiquan Zheng

USA

Najia Hajjaj-Hassouni

Morocco

Nam-Jong Paik

South Korea

Naomi Kobayashi

Japan

Nasser Al-Daghri

Saudi Arabia

Navin Fernando

USA

Neal Chen

USA

Neeraj Garg

UK

Nefyn Williams

UK

Negin Mohtasham

Iran

Neil Sheth

USA

Nens Van Alfen

Netherlands

Nevsun Inanc

Turkey

Nevzat Selim Gokay

Turkey

Niamh Moloney

Australia

Nicholas Henschke

Germany

Nicholas Clement

UK

Nicholas Chew

Singapore

Nicola Fazio

Italy

Nicola Walsh

UK

Nicolo' Martinelli

Italy

Nigel Morrison

Australia

Nikolaos Gougoulias

UK

Nilay Sahin

Turkey

Nils Bömer

Netherlands

Ning-Hung Chen

Taiwan

Nobuhiro Kamiya

USA

Noelle Junod Perron

Switzerland

Nur Kesiktas

Turkey

Oeystein Skare

Norway

Ole Marius Ekeberg

Norway

Olimpio Galasso

Italy

Oliver Faude

Switzerland

Olof Sköldenberg

Sweden

Olufemi Ayeni

Canada

Onno G. Meijer

Netherlands

Osmo Tervonen

Finland

Ozcan Konur

Turkey

Pablo Herrero

Spain

Panagiotis Koulouvaris

Greece

Panos Thomas

UK

Paolo Pillastrini

Italy

Paresh Jobanputra

UK

Parshia Moghadas

UK

Pascal Madeleine

Denmark

Pascal Richette

France

Pasquale Farsetti

Italy

Patric Garcia

Germany

Patrick O'Toole

Ireland

Patrizio Sale

Italy

Paul Hendrick

UK

Paul Arnold

USA

Paul Kuzyk

Canada

Paul Orrock

Australia

Paul Kaloostian

USA

Paul C Willems

Netherlands

Paul Jarle Mork

Norway

Paula Korn

Germany

Paulien Roos

UK

Pedro Labronici

Brazil

Per Kjaer

Denmark

Per Aspenberg

Sweden

Pericles Papadopoulos

Greece

Pernilla Eliasson

Sweden

Peter Leggat

Australia

Peter Pilot

Netherlands

Peter Oesch

Switzerland

Peter Angele

Germany

Peter Junker

Denmark

Peter Augat

Germany

Peter Michael Kent

Denmark

Petri Salo

Finland

Petteri Hovi

Finland

Ph Chau

Hong Kong

Phil Page

USA

Philip Van Der Wees

Netherlands

Philip Robinson

Australia

Philip Kasten

Germany

Philip Stahel

USA

Philippe Terrier

Switzerland

Pier Maria Fornasari

Italy

Pierre Hepp

Germany

Pierre Langevin

Canada

Pietro Spennacchio

Italy

Pilar Escolar-Reina

Spain

Ping Sun

USA

Pol Maria Rommens

Germany

Prasad Gourineni

USA

Przemyslaw pdowski

Poland

Qing Jiang

China

Qi-Rong Qian

China

Quinette Louw

South Africa

Rachael Gooberman-Hill

UK

Rachelle Buchbinder

Australia

Rad Zdero

Canada

Rafael Pinto

Australia

Rafael Lomas-Vega

Spain

Rahman Shiri

Finland

Raimon Sanmarti

Spain

Raimund Wagener

Germany

Raine Sihvonen

Finland

Rajesh Rohilla

India

Raju Vaishya

India

Ran Nakashima

Japan

Rana Hinman

Australia

Randy Mascarenhas

Canada

Ranjit Lall

UK

Raphael Catane

Israel

Raphael Adobor

Norway

Rebecca Moon

UK

Reinhard Schnettler

Germany

Remko Soer

Netherlands

Rene Fejer

Denmark

Rene Hartensuer

Germany

Renee Stark

Germany

Riccardo Del Vescovo

Italy

Richa Gupta

India

Richard Martin

USA

Richard Mannion

UK

Richard Loeser

USA

Richard Stange

Germany

Richard Bohannon

USA

Richard Crist

USA

Richard Schaefer

USA

Rieke Alten

Germany

Rita Durigan

Brazil

Robert Adler

USA

Robert Barkin

USA

Robert Terkeltaub

USA

Roberta Ramonda

Italy

Roberta Bonfiglioli

Italy

Robin Dore

USA

Roger Van Riet

Belgium

Rogeria Serakides

Brazil

Rolf Burghardt

Germany

Ronald Van Vollenhoven

Sweden

Rong Mu

China

Rong Song

China

Rosaria Guzzo

USA

Rosely Godinho

Brazil

Ross Iles

Australia

Rossana Scrivo

Italy

Rüdiger Junker

Germany

Ruofu Zhu

China

Samuel Bederman

USA

Saad Nseir

France

Sabine Ochman

Germany

Saeideh Mazloomzadeh

Iran

Salih Ozgocmen

Turkey

Sally Wilson

UK

Sally Roberts

UK

Salvatore Minisola

Italy

Salvi Prat

Spain

Sami Kolta

France

Samuel W Cadden

UK

Sandro Giannini

Italy

Sanna Mari Kääriä

Finland

Sara Checa

Germany

Sarah Zawodny

USA

Sarah Kingsbury

UK

Sascha Colen

Belgium

Scott Lovald

USA

Sda Sda

Armenia

Sean Waldron

USA

Sebastian Farr

Austria

Seiya Jingushi

Japan

Senthil Nathan Sambandam

India

Sergio Cunha

Brazil

Sergio Munoz

Chile

Sergio Vargas-Prada

Spain

Serkan Inceoglu

USA

Seyed Mohsen Mir

Iran

Shabir Dhar

India

Shahril Shaarani

Ireland

Shannon Hughes

USA

Shaun O'Leary

Australia

Shaw-Ruey Lyu

Taiwan

Shea Palmer

UK

Sheree Nix

Australia

Shianchao Tay

Singapore

Shigeru Kobayashi

Japan

Shigeyuki Muraki

Japan

Shingo Maeda

Japan

Shiro Ikegawa

Japan

Shital Parikh

USA

Shuang Wang

China

Shu-Guang Gao

China

Shuman Yang

USA

Shunichiro Okazaki

Japan

Shun-Jen Chang

Taiwan

Sibylle Grad

Switzerland

Siddhartha Sikdar

USA

Sigurd Liavaag

Norway

Sihyun Kim

South Korea

Silvia Gianola

Italy

Simo Saarakkala

Finland

Simon French

Australia

Simon Kollnberger

UK

Simon Yeung

Hong Kong

Sionnadh Mclean

UK

Slawomir Snela

Poland

Sofia Ramiro

Netherlands

Sofia Chatziioannou

Greece

Sohail Mirza

USA

Sokichi Maniwa

Japan

Soren Thorgaard Skou

Denmark

Spiros Pneumaticos

Greece

Srinath Kamineni

USA

Stacie Salsbury

USA

Stefano Zaffagnini

Italy

Stefano Campi

Italy

Stefano Negrini

Italy

Stephanie Muth

USA

Stephen Richardson

UK

Stephen Perle

USA

Stephen Alway

USA

Stephen Lane

Australia

Stephen Badylak

USA

Stephen May

UK

Steven Kamper

Australia

Stewart Leavitt

USA

Stig Storgaard Jakobsen

Denmark

Suhail Chaudhry

UK

Suleyman Koca

Turkey

Suppasin Soontrapa

Thailand

Surya Bhan

India

Susan Iannaccone

USA

Susan Kurrle

Australia

Susan Roush

USA

Susan Grimston

USA

Su-Yin Yang

Singapore

Suzanne Guerin

Ireland

Sylvia Von Mackensen

Germany

Tim Schepers

Netherlands

Tae-Joon Cho

South Korea

Taís Malysz

Brazil

Takashi Nagai

USA

Takefumi Furuya

Japan

Takehiko Matsushita

Japan

Takeshi Morii

Japan

Takui Ito

Japan

Takuma Yamasaki

Japan

Tania Winzenberg

Australia

Tanja Nauck

Germany

Tara Brennan-Speranza

Australia

Tasha Stanton

Australia

Teguh Haryo Sasongko

Malaysia

Theodoros B. Grivas

Greece

Thomas Gardner

USA

Thomas Jakobsen

Denmark

Thomas Heyse

Germany

Thomas Hoogeboom

Netherlands

Thomas Le Corroller

France

Thomas Andersen

Denmark

Thomas Mittlmeier

Germany

Thomas Tischer

Germany

Thomas Quinn

Canada

Thomas Viskum Gjelstrup Bredahl

Denmark

Tina Juul

Denmark

Ting Xia

USA

Todd Davenport

USA

Tohr Nilsson

Sweden

Tom Van Raaij

Netherlands

Tom Gordon

Australia

Tomi Mäkinen

Finland

Tommaso Bonanzinga

Italy

Tommaso Pizzorusso

Italy

Tomohiro Onodera

Japan

Tomokazu Yoshioka

Japan

Tony Bateman

UK

Toru Maruyama

Japan

Toru Moro

Japan

Toshiaki Takahashi

Japan

Toshihisa Kojima

Japan

Tsae-Jyy Wang

Taiwan

Tsukasa Sakurada

Japan

Tuan Nguyen

Australia

Ukadike Chris Ugbolue

UK

Utku Kandemir

USA

Vanessa Yingling

USA

Victor Hoe

Malaysia

Victoria Pennick

Canada

Vijay Goel

USA

Vikas Agarwal

India

Vipul Vijay

India

Vivian Teixeira

Brazil

Wael Muselmani

Syria

Wanda Forczek

Poland

Weiya Zhang

UK

Wenlan Wu

Taiwan

Werner Marx

Germany

Werner Kolb

Germany

Werner Schmoelz

Austria

Wilbert Van Den Hout

Netherlands

Wilfried Mau

Germany

William Shaw

USA

Win-Ping Deng

Taiwan

Wolfgang Viechtbauer

Netherlands

Worawit Louthrenoo

Thailand

Wouter Den Hollander

Netherlands

X. Lucas Lu

USA

Xavier Chevalier

France

Xenofon Baraliakos

Germany

Xia Qun

China

Xing Zhao

USA

Xinghuo Wu

China

Xingzhong Jin

Australia

Xiuchun Yu

China

Xu Shengqian

China

Yohan Robinson

Sweden

Yong Ahn

South Korea

Yong-Chan Ha

South Korea

Yoon Hyuk Kim

South Korea

Yoshiyuki Hakeda

Japan

Yuanyuan Wang

Australia

Yufeng Dong

USA

Yuichi Kasai

Japan

Yulong Sun

China

Yves Renaudineau

France

Yves Henchoz

Canada

Yves Henrotin

Belgium

Zafar Fatmi

Pakistan

Zhan-Jun Shi

China

Zhen Li

Switzerland

Zhou Xiang

China

Zinon Kokkalis

Greece

Zong Mei

Sweden

## Pre-publication history

The pre-publication history for this paper can be accessed here:

http://www.biomedcentral.com/1471-2474/15/15/prepub

